# Supplementary material for: Chemical, Physical and Biological Triggers of Evolutionary Conserved Bcl-xL-Mediated Apoptosis
Source: Cancers (Basel). 2020 Jun 25;12(6):1694. doi: 10.3390/cancers12061694 (PMC7352625; doi:10.3390/cancers12061694)
Supplement: Supplementary file 1 [file cancers-12-01694-s001.zip › Westerns.pptx]

## Slide 1
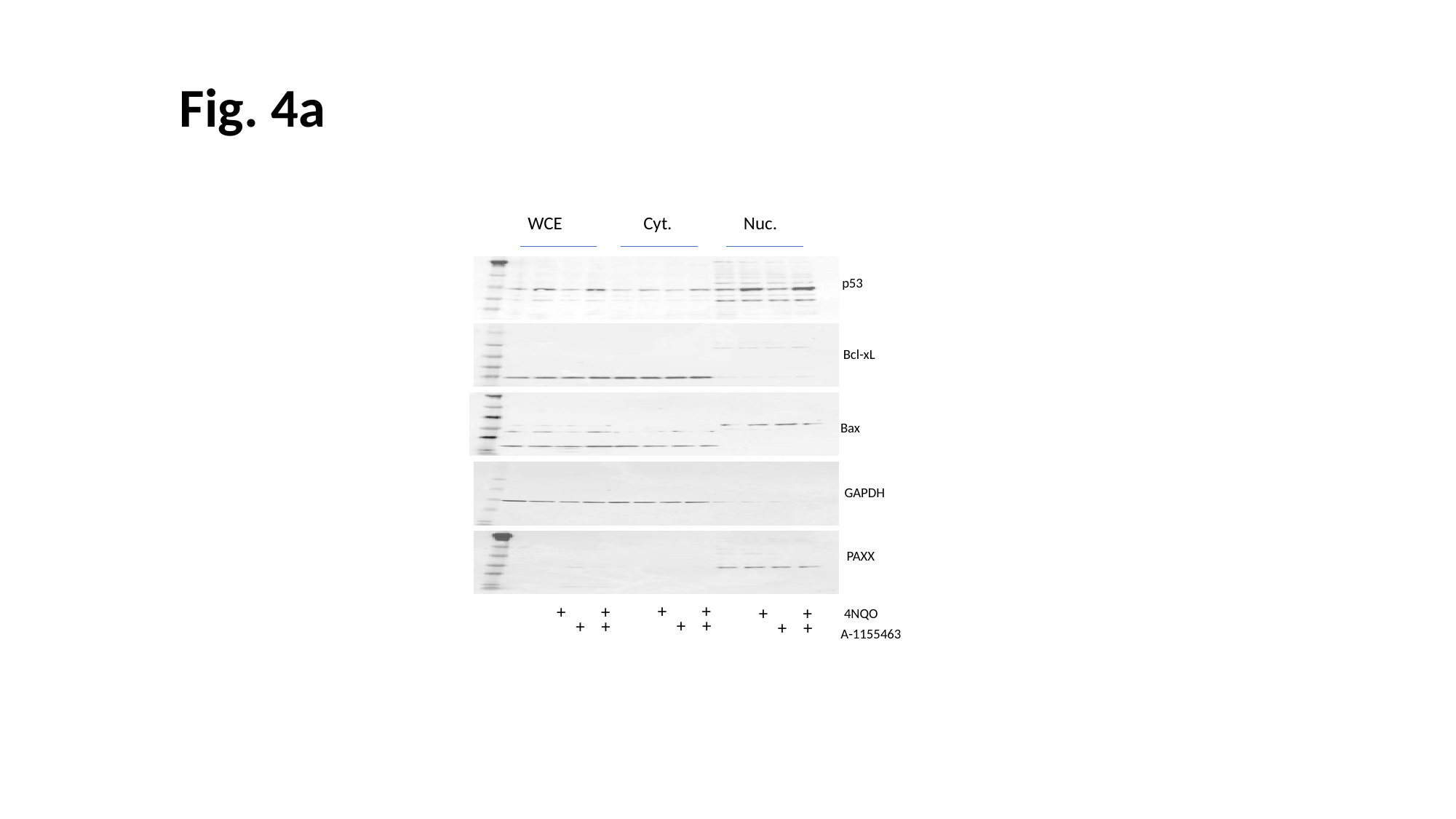

Fig. 4a
WCE
Cyt.
Nuc.
p53
Bcl-xL
Bax
GAPDH
PAXX
+
+
+
+
+
+
4NQO
+
+
+
+
+
+
A-1155463

## Slide 2
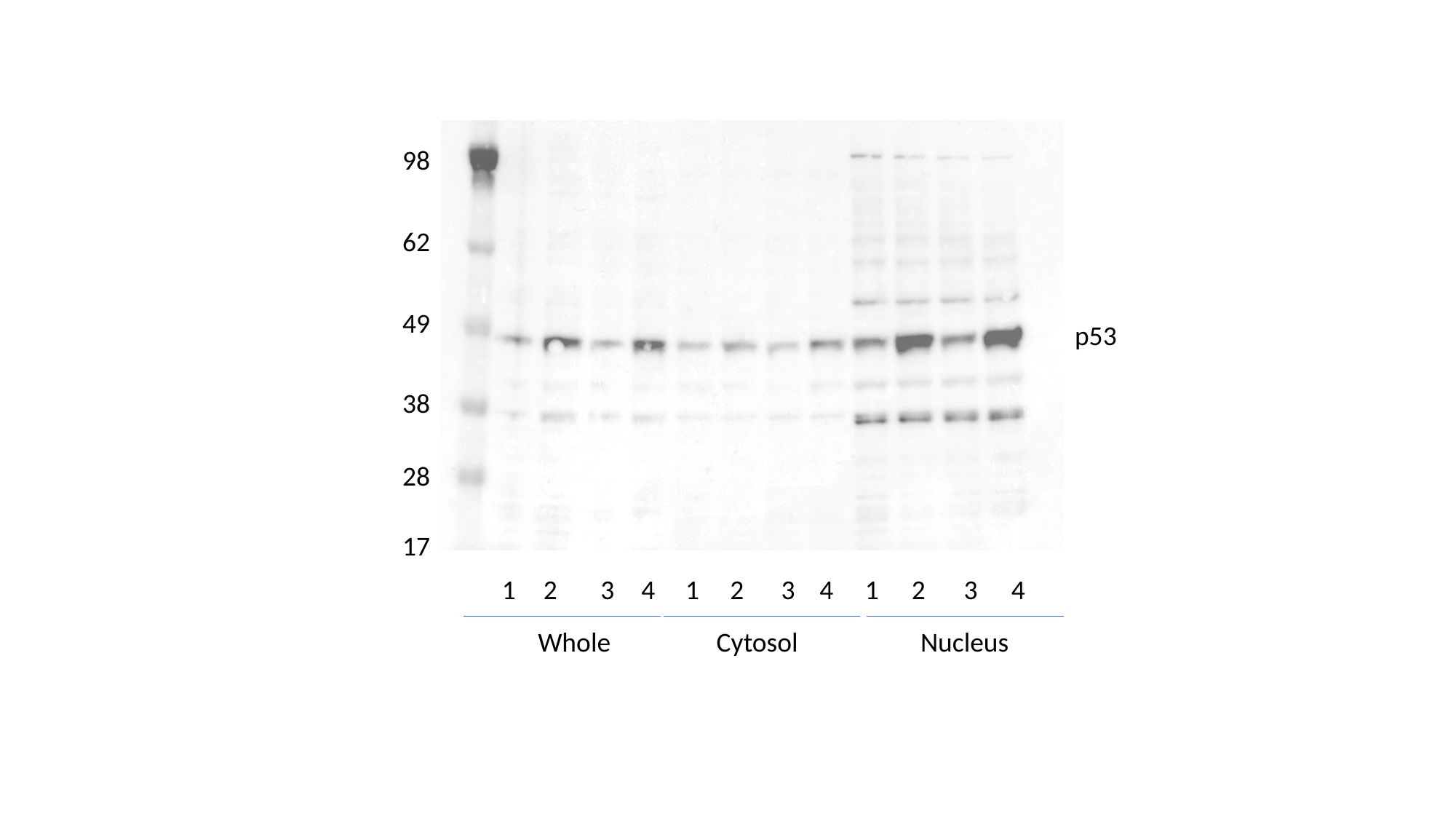

98
62
49
p53
38
28
17
1
2
3
4
1
2
3
4
1
2
3
4
Whole
Cytosol
Nucleus

## Slide 3
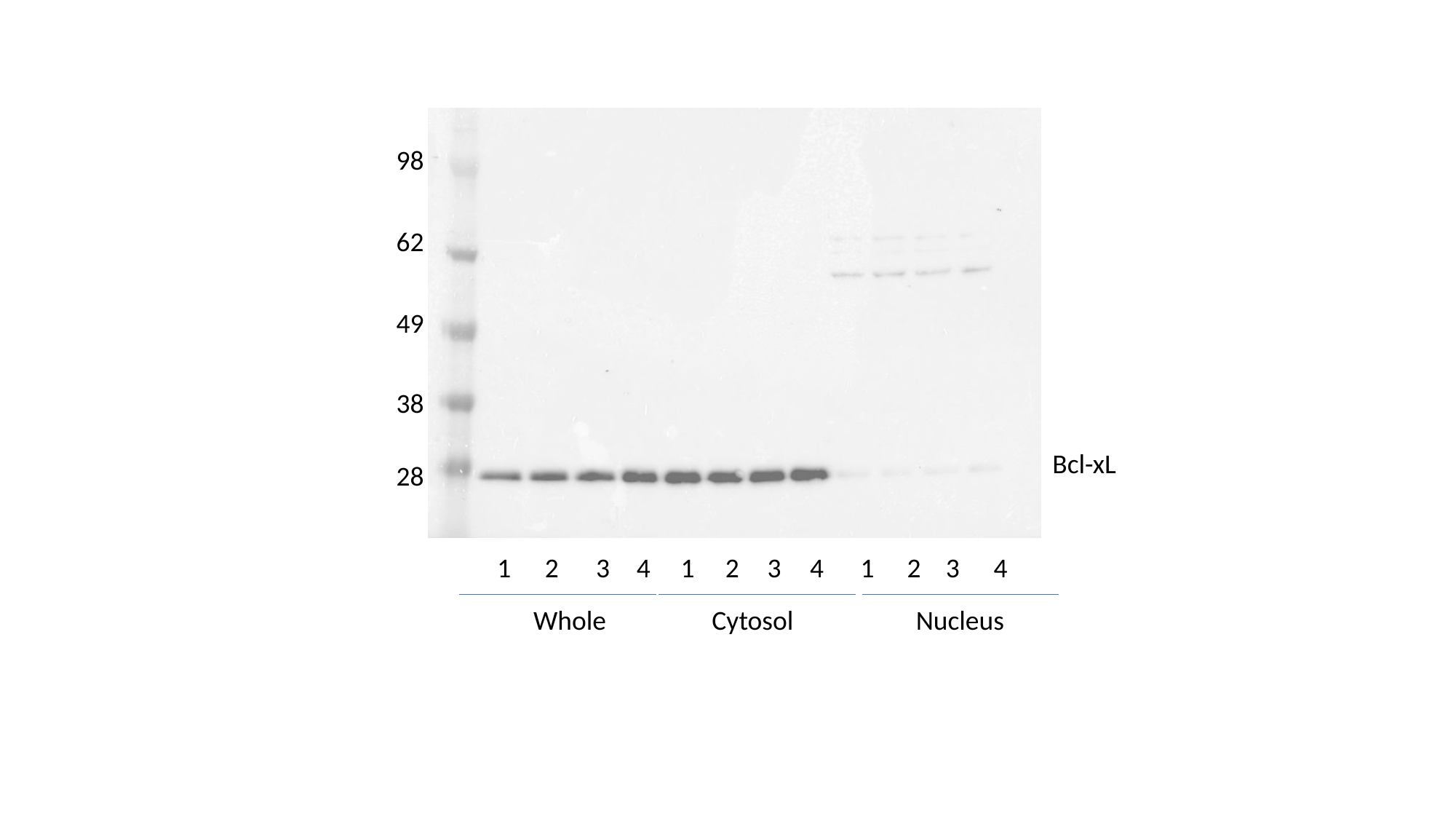

98
62
49
38
Bcl-xL
28
1
2
3
4
1
2
3
4
1
2
3
4
Whole
Cytosol
Nucleus

## Slide 4
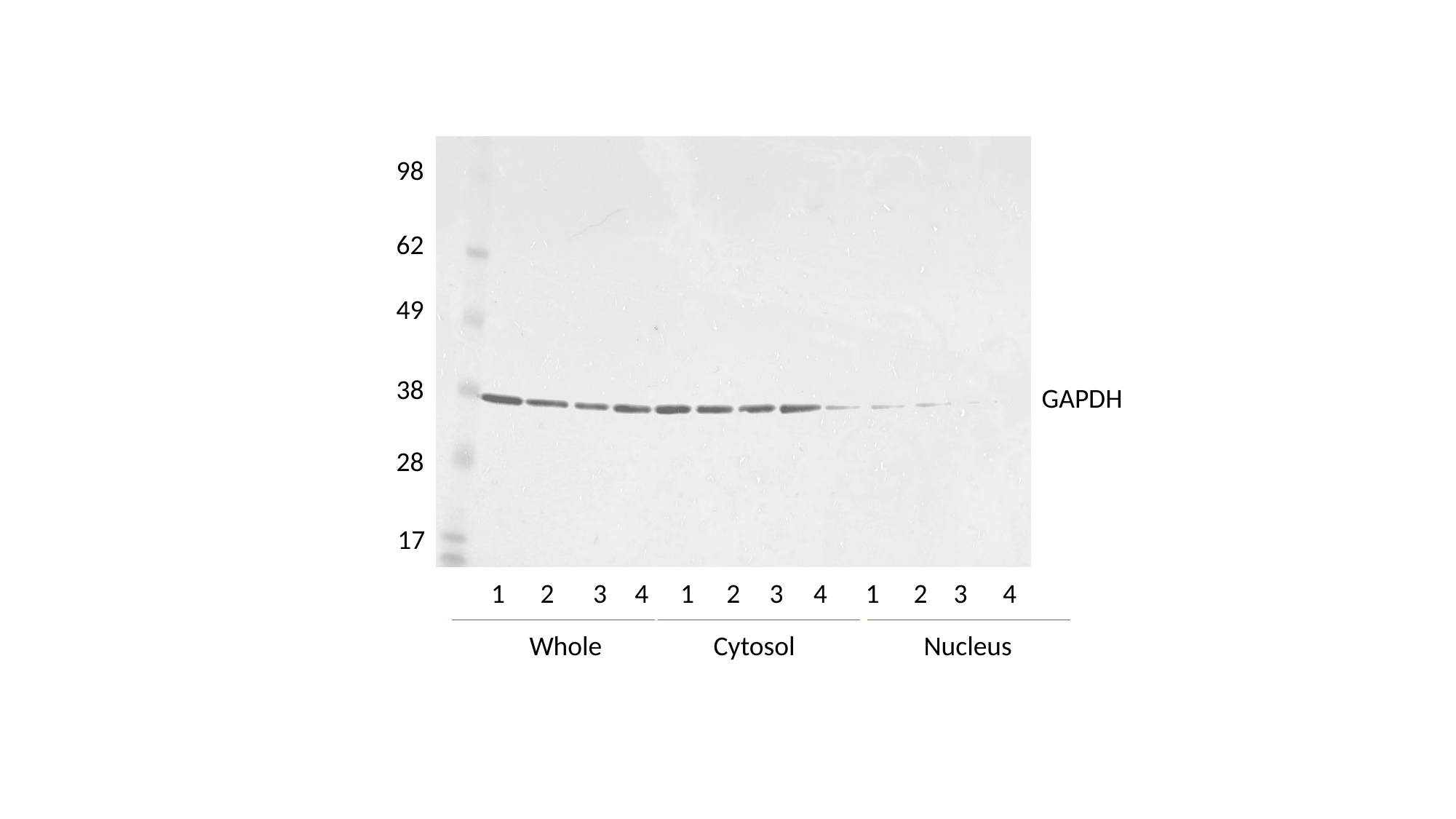

98
62
49
38
GAPDH
28
17
1
2
3
4
1
2
3
4
1
2
3
4
Whole
Cytosol
Nucleus

## Slide 5
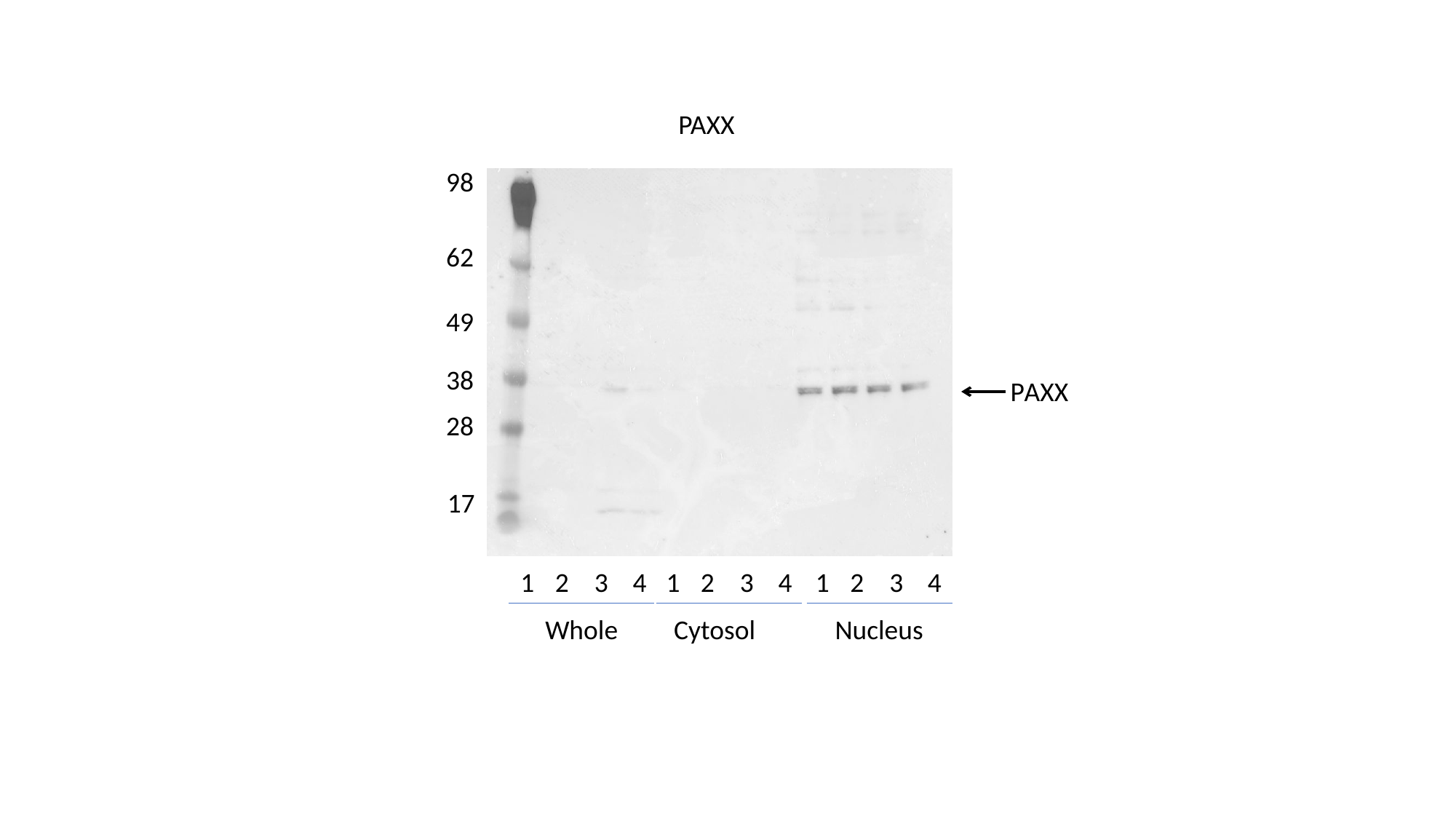

PAXX
98
62
49
38
PAXX
28
17
1
2
3
4
1
2
3
4
1
2
3
4
Whole
Cytosol
Nucleus

## Slide 6
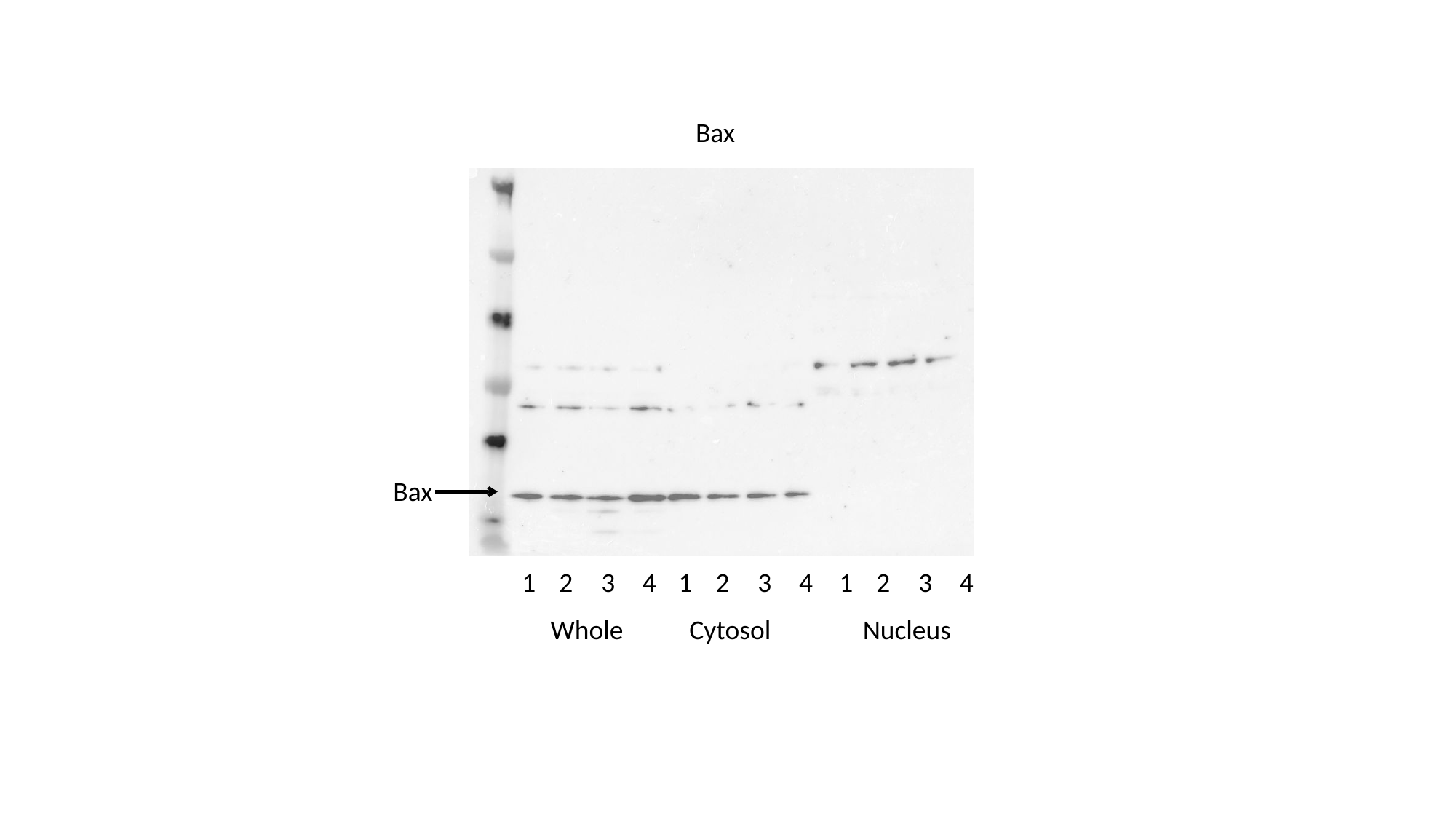

Bax
Bax
1
2
3
4
1
2
3
4
1
2
3
4
Whole
Cytosol
Nucleus

## Slide 7
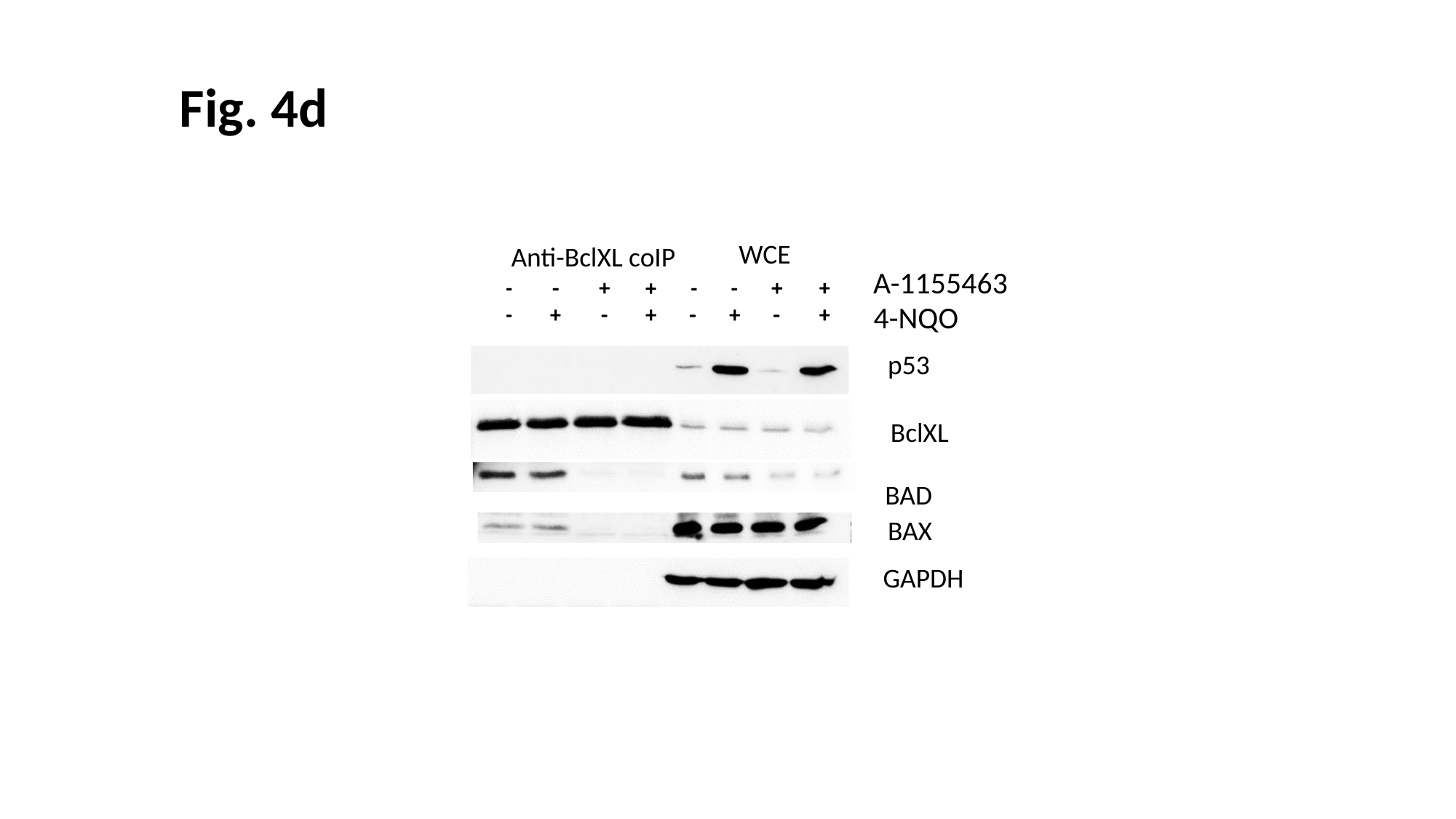

Fig. 4d
WCE
Anti-BclXL coIP
A-1155463
4-NQO
p53
BclXL
BAD
BAX
GAPDH

## Slide 8
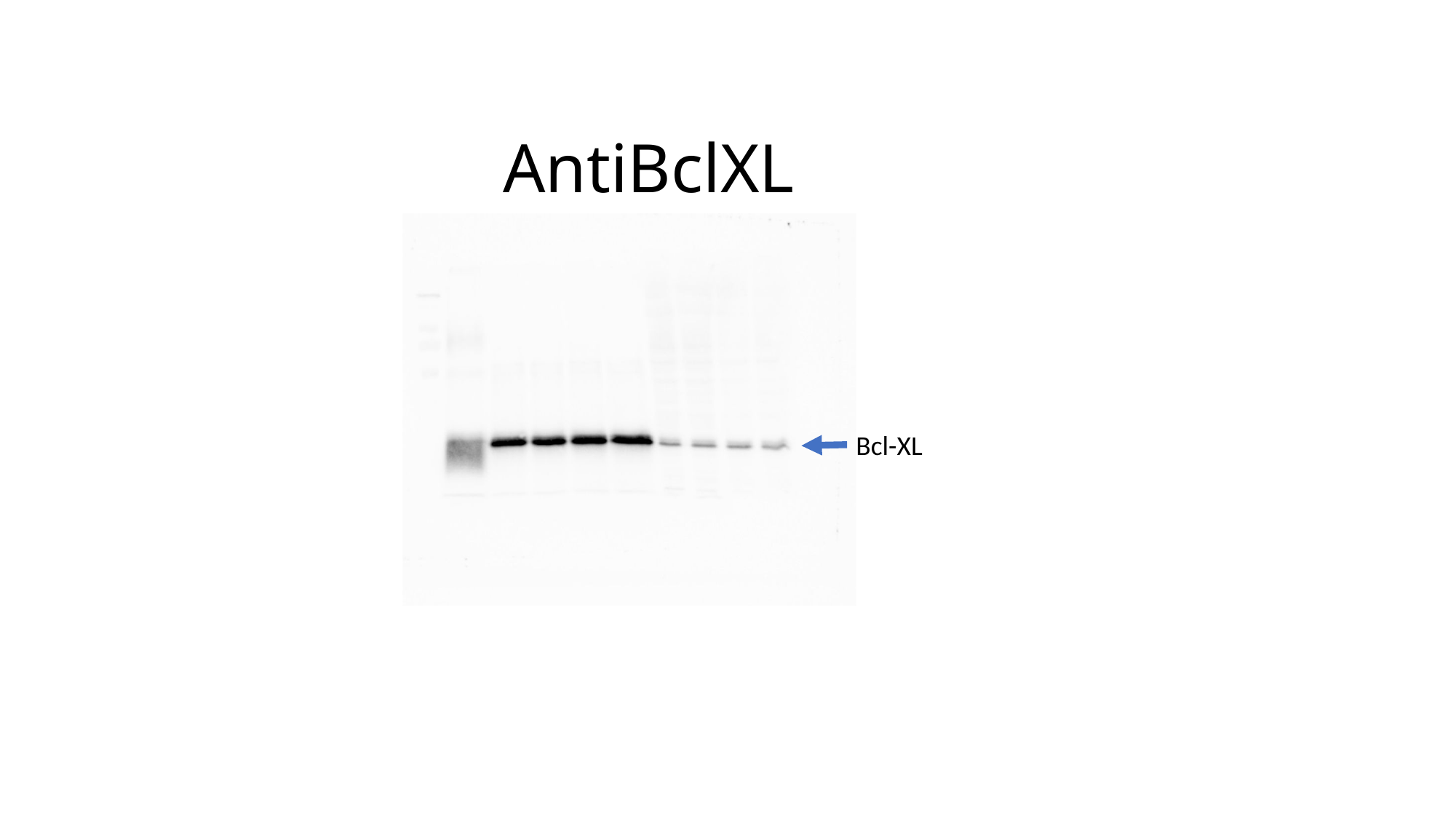

# AntiBclXL
Bcl-XL

## Slide 9
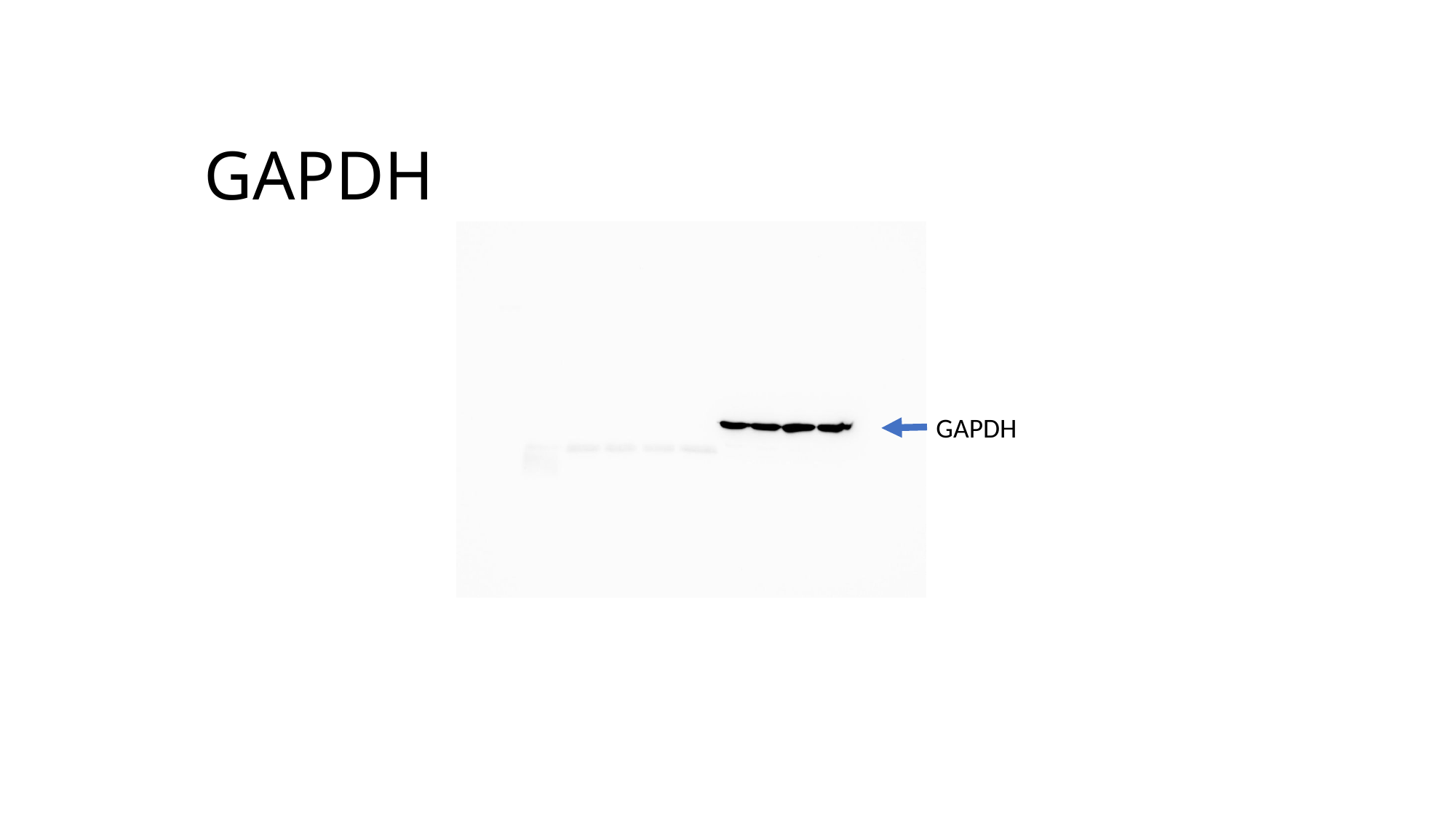

# GAPDH
GAPDH

## Slide 10
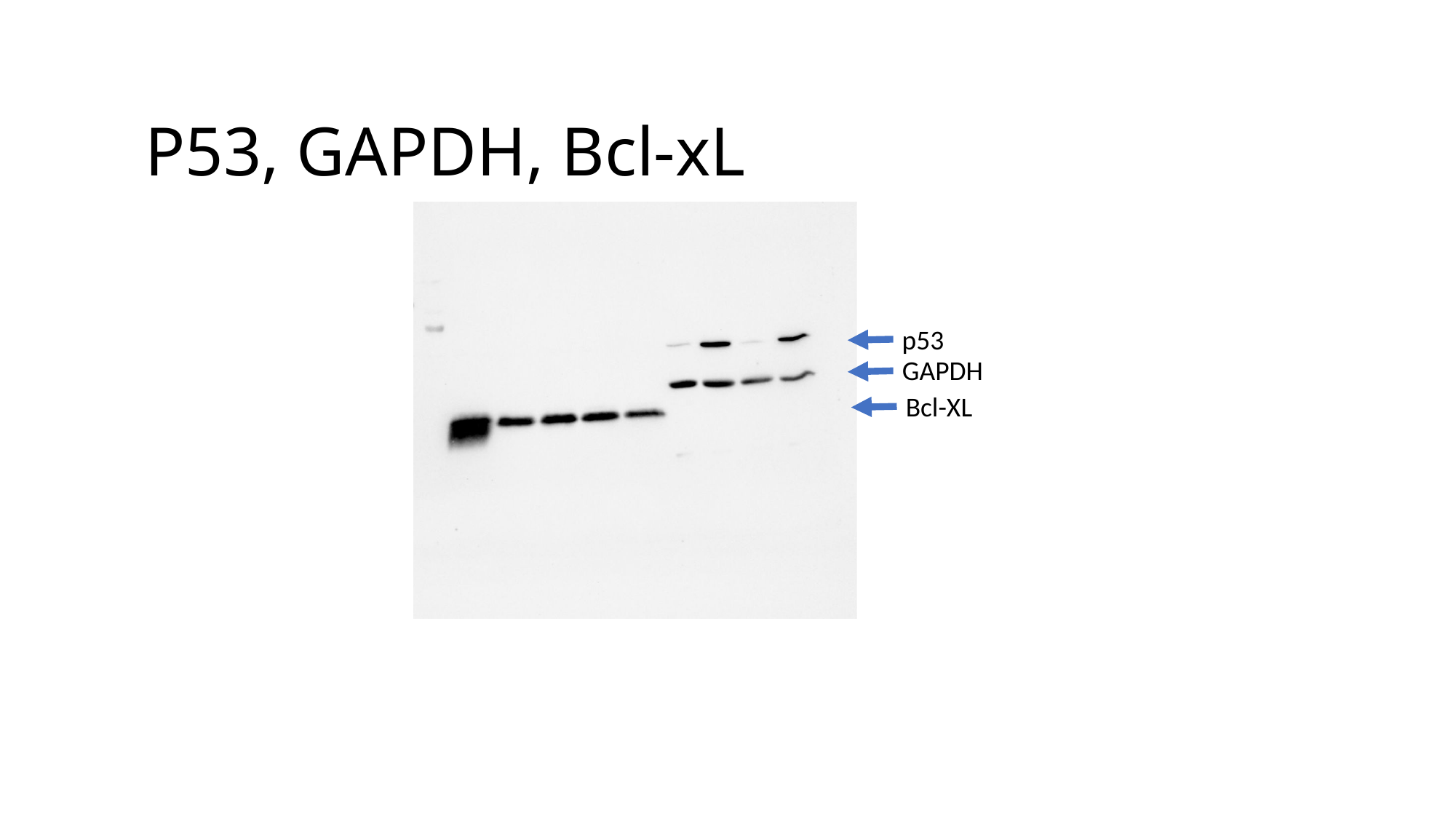

# P53, GAPDH, Bcl-xL
p53
GAPDH
Bcl-XL

## Slide 11
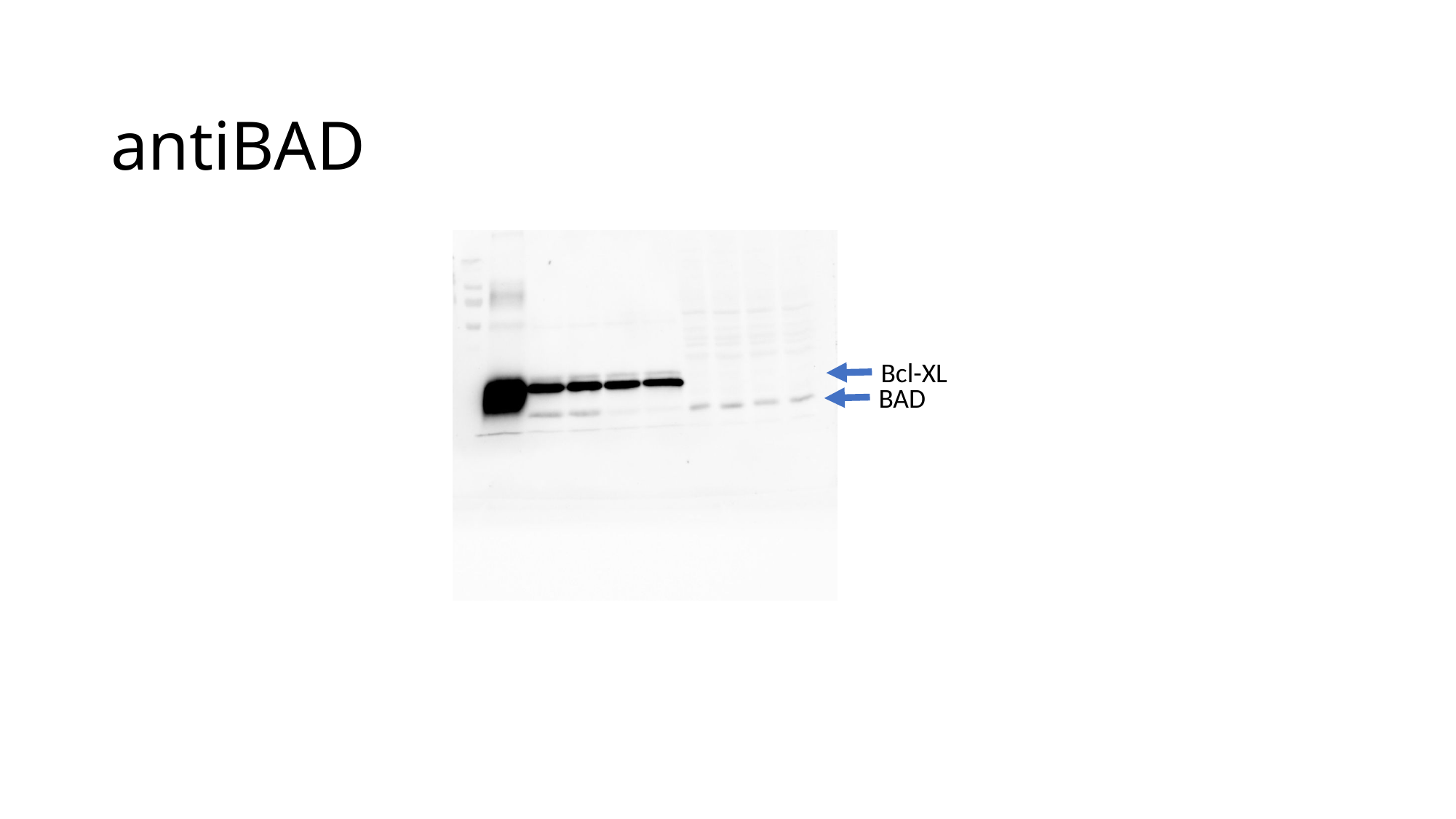

# antiBAD
Bcl-XL
BAD

## Slide 12
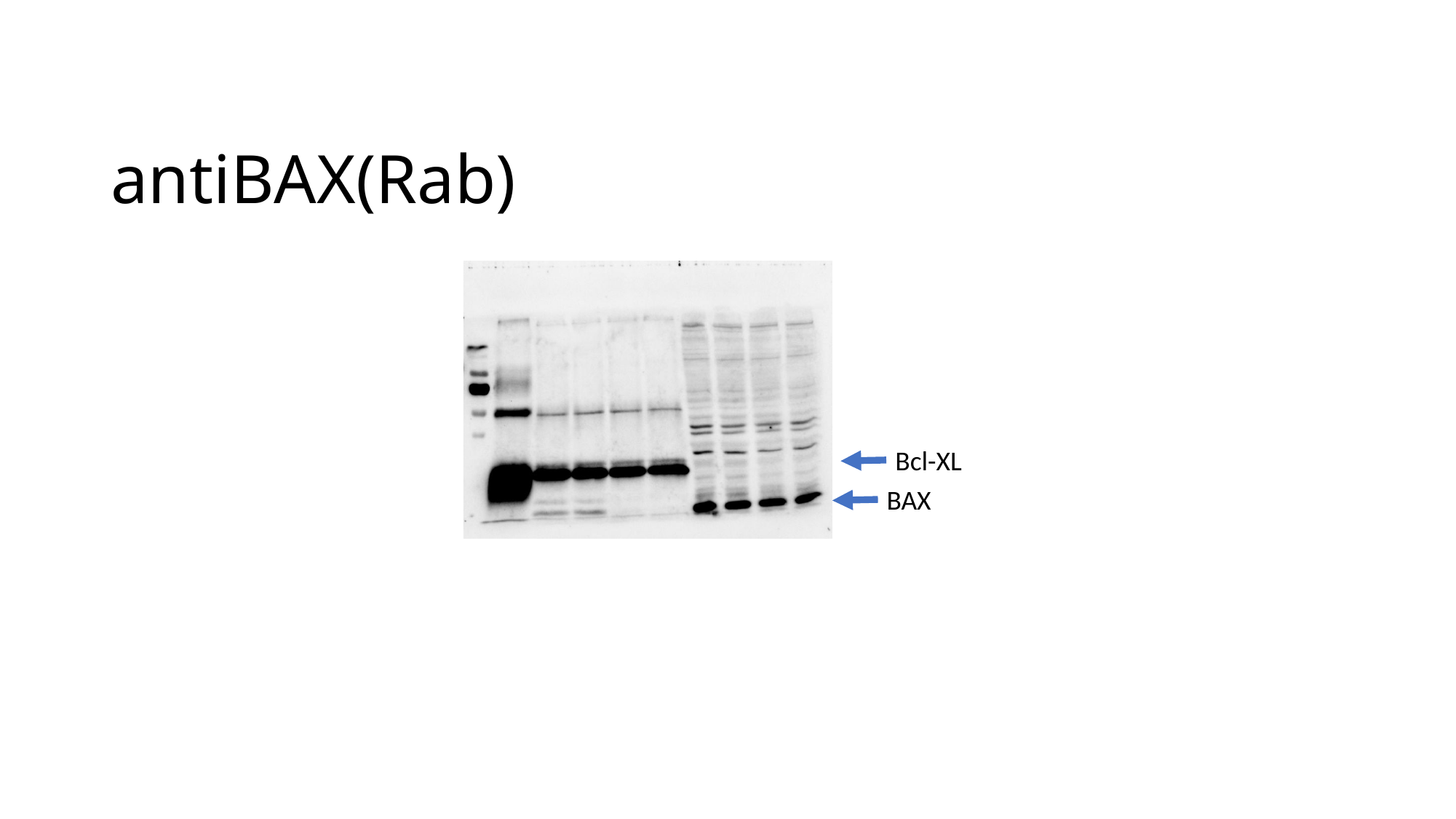

# antiBAX(Rab)
Bcl-XL
BAX

## Slide 13
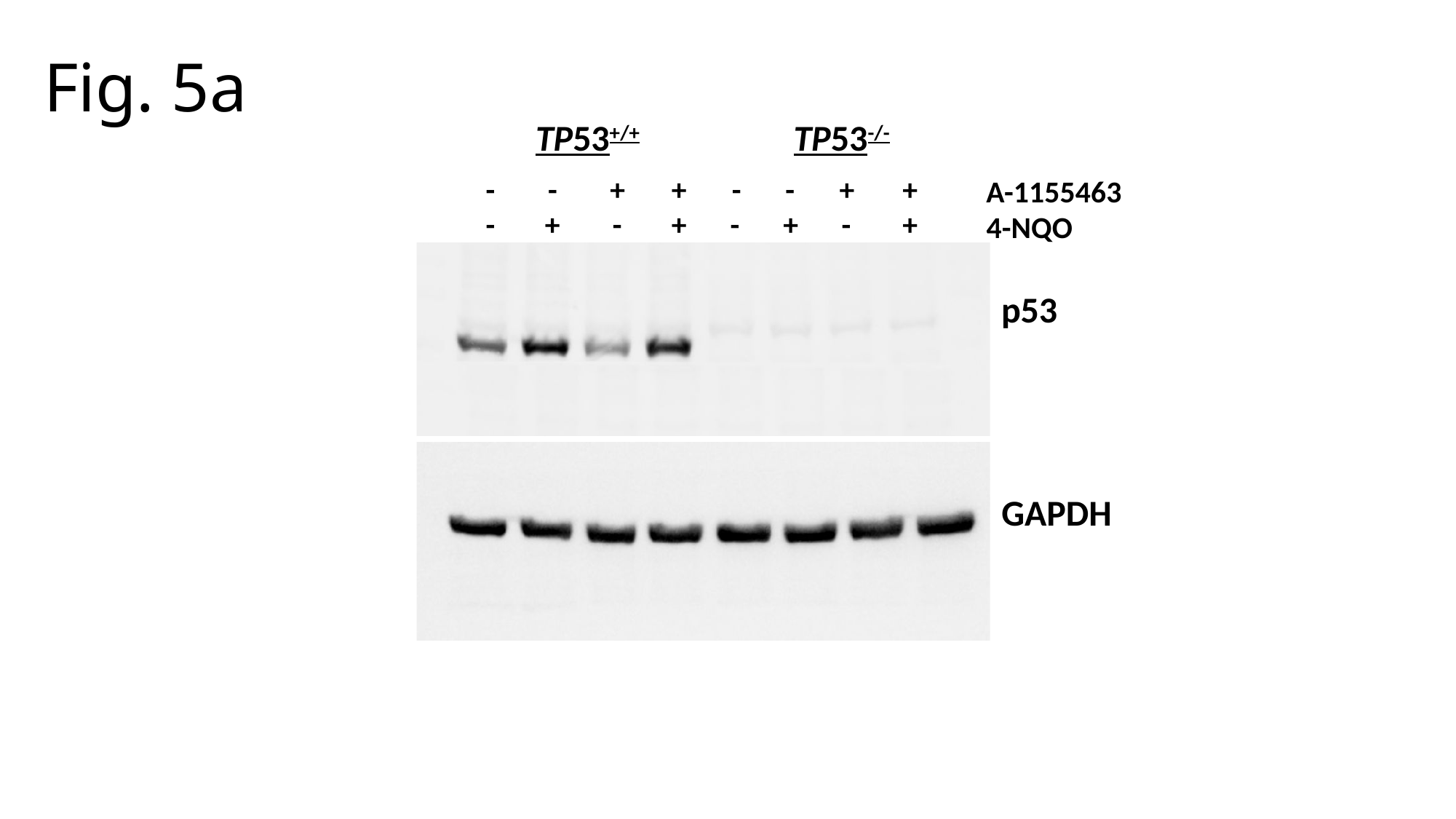

# Fig. 5a
TP53+/+
TP53-/-
-
-
+
-
-
+
+
+
A-1155463
-
+
-
+
-
+
-
+
4-NQO
p53
GAPDH

## Slide 14
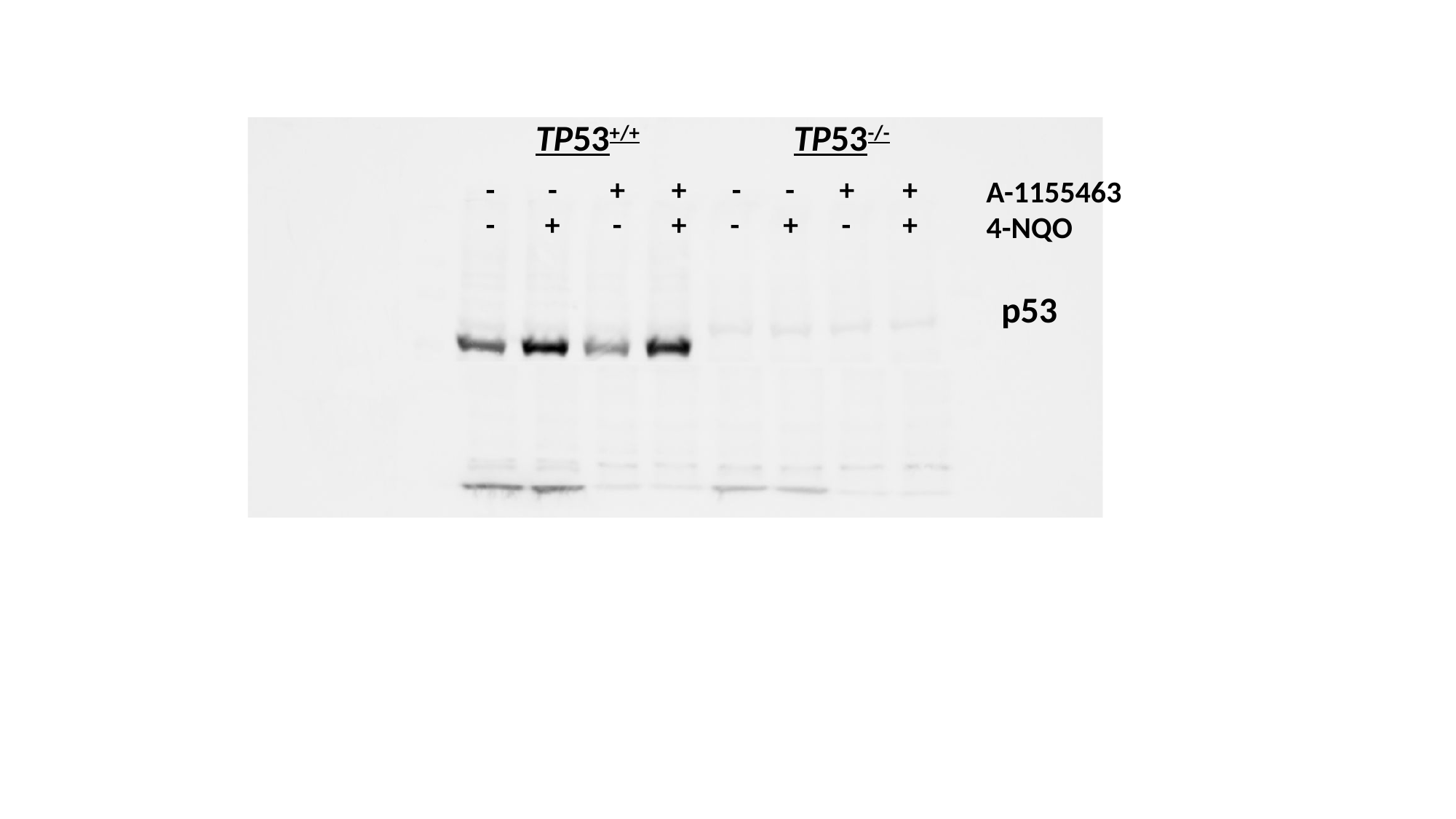

TP53+/+
TP53-/-
-
-
+
-
-
+
+
+
A-1155463
-
+
-
+
-
+
-
+
4-NQO
p53

## Slide 15
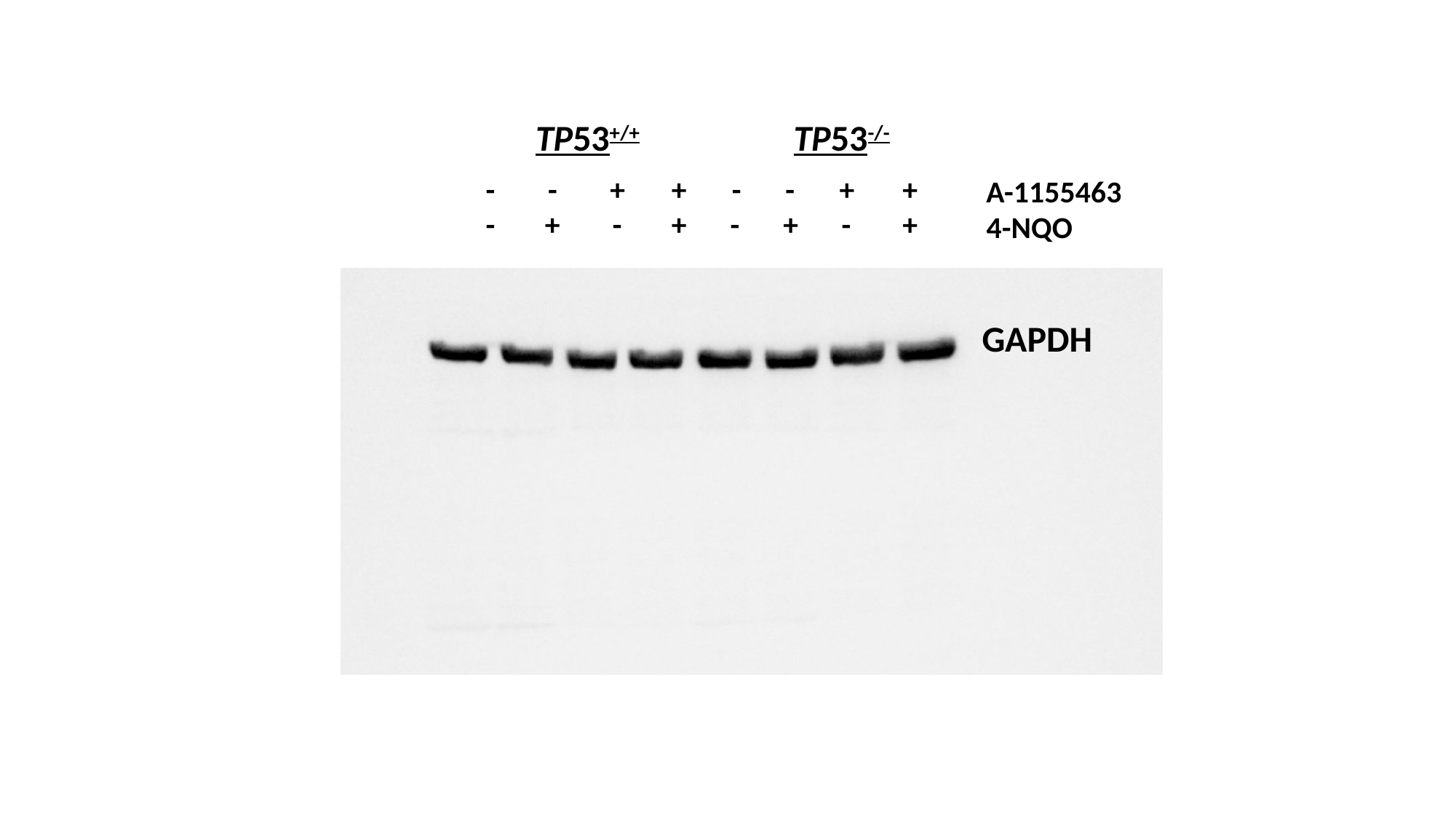

TP53+/+
TP53-/-
-
-
+
-
-
+
+
+
A-1155463
-
+
-
+
-
+
-
+
4-NQO
GAPDH

## Slide 16
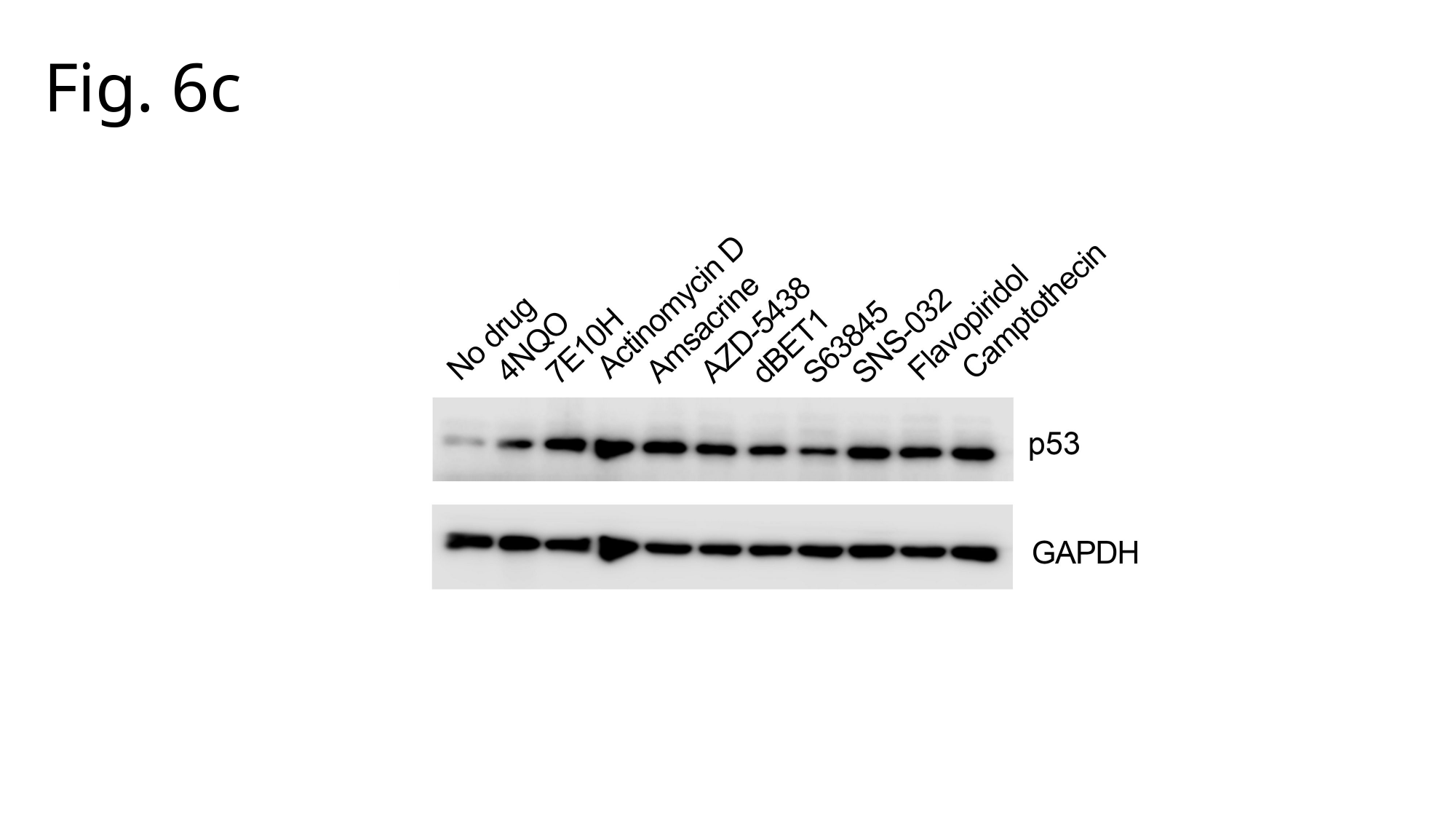

# Fig. 6c

## Slide 17
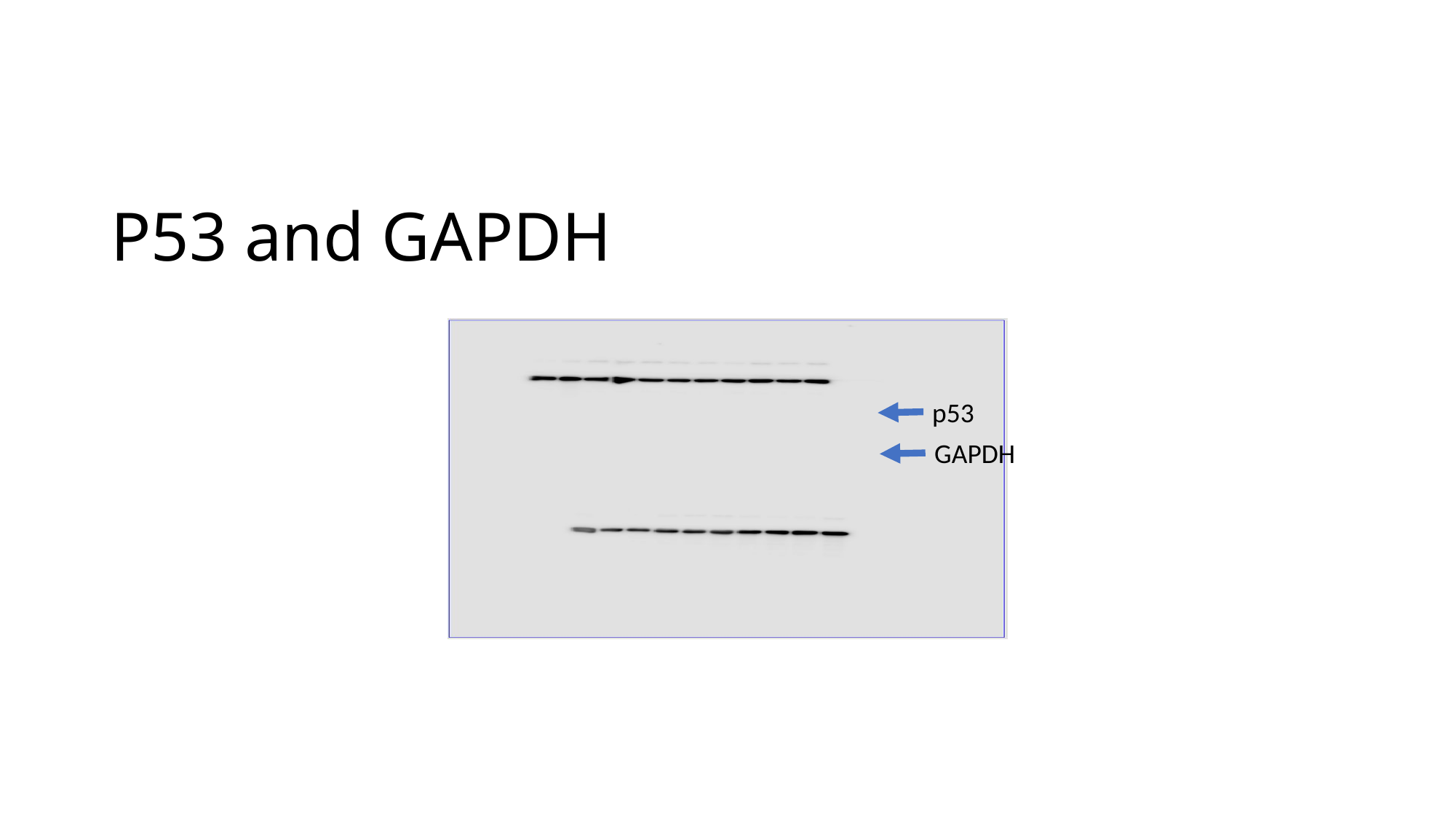

# P53 and GAPDH
p53
GAPDH

## Slide 18
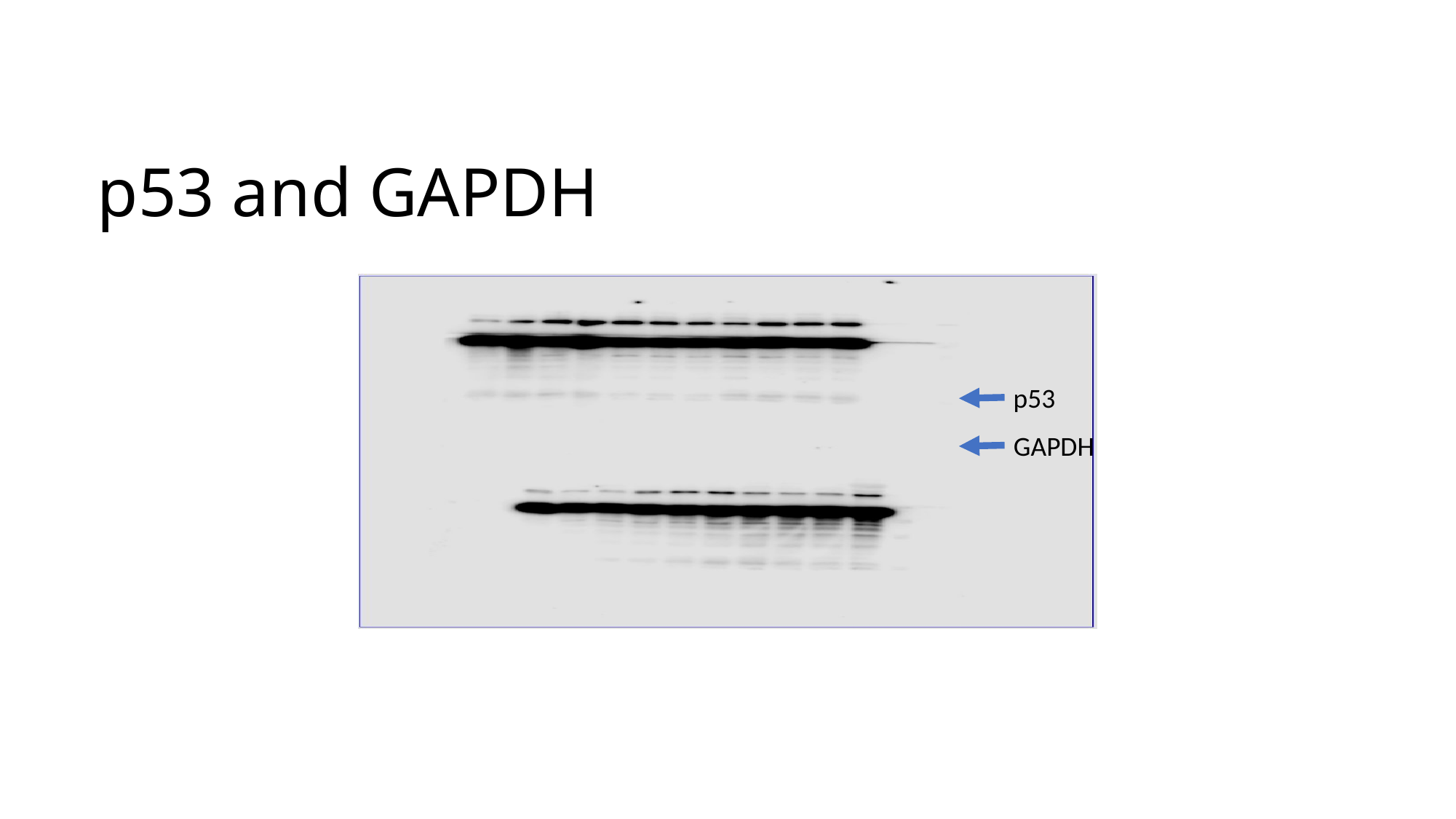

# p53 and GAPDH
p53
GAPDH
